# Rapid Screening of Early SARS-CoV-2 Variants of Concern by Pyrosequencing

**DOI:** 10.3390/ijms27219769

**Published:** 2026-10-30

**Authors:** Susanne C. Duwe, Ashkan Ghassemi, Sofia Paraskevopoulou, Jeanette Milde, Thorsten Wolff, Max von Kleist, Ralf Dürrwald, Martin Hölzer, Djin-Ye Oh

**Affiliations:** 1 Unit 17: Influenza and Other Respiratory Viruses, Robert Koch Institute, 13353 Berlin, Germany; duwes@rki.de (S.C.D.); mildej@rki.de (J.M.); wolfft@rki.de (T.W.); duerrwaldr@rki.de (R.D.); 2 Genome Competence Center (MF1), Robert Koch Institute, 13353 Berlin, Germany; ashkan.ghassemi@charite.de (A.G.); paraskevopoulous@rki.de (S.P.); hoelzerm@rki.de (M.H.); 3 Molecular Epidemiology, Berlin Institute of Health, Charité Universitätsmedizin Berlin, 10178 Berlin, Germany; 4 Project Group 5 (P5): Systems Medicine of Infectious Diseases, Robert Koch Institute, 13353 Berlin, Germany; kleistm@rki.de; 5 Department of Mathematics and Computer Science, Freie Universität Berlin, 14195 Berlin, Germany

**Keywords:** SARS-CoV-2, pyrosequencing, spike gene, variants of concern, rapid identification, SARS-CoV-2 VOC screening, COVID-19 surveillance, genome surveillance, molecular diagnostics, virus mutations

## Abstract

SARS-CoV-2 variants of concern (VOCs) emerged in late 2020 and were characterized by increased transmissibility, virulence, or antigenic drift. Rapid identification in diagnostic materials is essential for effective implementation of public health responses. The spike gene, essential in viral entry and immune escape, frequently harbors VOC-specific mutations. Their detection by conventional sequencing methods remains time-consuming. A nested RT-PCR-based pyrosequencing (PSQ) assay was designed targeting three mutation-dense regions of the SARS-CoV-2 spike gene. Analytical specificity was assessed using defined controls. Clinical validation involved 175 SARS-CoV-2-positive specimens from the national German acute respiratory infections (ARI) sentinel covering Alpha, Beta, and Delta variants, with different subsets of spike mutations analyzed by PSQ. PSQ generated interpretable pyrograms and enabled reliable detection of predefined VOC-associated spike mutations. Direct comparison of PSQ calls with the corresponding WGS-derived sequences showed 98.6% concordance (489/496 evaluable comparisons). The workflow enabled same-day mutation profiling directly from diagnostic material, providing rapid sequence-level information on mutations associated with increased transmissibility and immune escape. These findings demonstrate that PSQ provides rapid and reliable screening of predefined spike mutations associated with early SARS-CoV-2 VOCs. The workflow may complement whole-genome sequencing in surveillance settings where rapid mutation-level information is required. In a broader sense, the study demonstrates a data-driven approach for developing and periodically adapting targeted molecular surveillance assays as viral populations evolve.

## 1. Introduction

SARS-CoV-2 variants of concern (VOCs) emerged repeatedly during the COVID-19 pandemic and shaped its course. Lineages such as Alpha, Beta, Gamma, Delta, and Omicron carried characteristic mutation constellations in the spike (S) gene that were associated with altered transmissibility, receptor binding, and susceptibility to neutralizing antibodies. The World Health Organization (WHO) classifies further lineages requiring enhanced surveillance as variants of interest (VOIs) or variants under monitoring (VUMs); VOCs represent the highest-risk category [1,2]. Because such genetic changes can alter viral fitness and population-level dynamics, rapid tools are needed to detect epidemiologically relevant mutations directly from clinical specimens [1,3]. The receptor-binding domain (RBD) is an important target for molecular surveillance because substitutions can affect receptor binding and antibody recognition, as demonstrated by deep mutational scanning [4,5]. Experimental studies of N501Y and of the Delta variant underscored the biological relevance of RBD mutations present in the early VOCs [6,7]. However, the detection of an individual substitution does not by itself establish a complete lineage assignment or a clinical phenotype.

Whole-genome sequencing (WGS) provides the resolution required to characterize viral evolution and distinguish circulating lineages and thus serves as the reference method for documenting variant emergence [8,9]. Although global sequencing activity has declined substantially relative to the peak of the COVID-19 pandemic, WGS remains the primary method for characterizing SARS-CoV-2 evolution, classifying lineages, and monitoring their circulation. However, its contribution to surveillance depends on sequencing capacity, turnaround time, and specimen ascertainment [10,11]. WGS requires specialized infrastructure and substantial resource allocation, resulting in turnaround times that are frequently incompatible with the necessity for real-time public health interventions during periods of heightened viral activity [8]. During the pandemic peak, WGS capacity was often insufficient to meet the demand for near-real-time variant identification, and many surveillance systems therefore introduced rapid, mutation-focused molecular assays alongside WGS [12,13,14,15,16,17,18,19]. While approaches such as allele-specific PCR with melting curve analysis and other approaches, e.g., targeted Sanger sequencing, enabled rapid screening for specific spike mutations, they were constrained by the prerequisite for frequent redesign and demonstrated variable performance metrics, particularly in specimens with low viral load or compromised quality [20].

Pyrosequencing (PSQ) offers a complementary approach for short-amplicon, mutation-focused applications. It provides sequence-resolved base calls at predefined nucleotide positions, is compatible with multi-well (e.g., 24-, 48-, or in older systems 96-well) formats, and allows rapid adaptation to emerging targets [21,22,23,24]. Because assay adaptation requires only primer redesign rather than generating new probe sets, PSQ can rapidly pivot to address shifting mutation targets. Despite these technical advantages, PSQ has been evaluated primarily in small clinical or diagnostic cohorts, rather than population-based surveillance settings [25,26]. Surveillance-derived specimens often undergo longer transport and storage times and are subject to more variable collection conditions than those in routine diagnostic workflows. This pre-analytical variability can lead to greater heterogeneity in viral load and sample quality, both of which are well-known factors that affect the performance of molecular and sequencing-based assays [21,23].

Germany’s national acute respiratory infections (ARI) sentinel system is a long-standing, well-established platform for monitoring respiratory viruses [22,23,27,28,29]. During the COVID-19 pandemic, surveillance through this system was expanded to include SARS-CoV-2. This enabled evaluation of variant detection tools and mutation-focused assays under routine surveillance conditions [2]. The ARI sentinel system operated under the constraints of the pandemic response, which included the need for high sample throughput and rapid data generation despite variable specimen quality [23].

In this study, a nested reverse-transcription PCR (RT-PCR) pyrosequencing assay targeting three mutation-dense regions of the SARS-CoV-2 spike gene was designed and evaluated. The design focused on receptor-binding domain (RBD) mutations characteristic of early circulating VOCs in Germany in 2021. We assessed analytical specificity using defined controls and, where available, compared PSQ mutation calls with whole-genome sequencing data from ARI sentinel specimens. The objective was not to establish PSQ as a method for identifying new variants or as a replacement for WGS, but rather to evaluate a mutation-focused surveillance approach for the rapid detection of predefined, epidemiologically relevant substitutions in routine sentinel specimens.

More generally, WGS at scale is often too slow and costly to inform public health decisions in real-time [30]. Viral evolution under immune and receptor-binding selection may converge on a limited number of sites. This convergence can be exploited, as focused assays deliver sequencing results faster and across a larger proportion of circulating specimens than routine WGS permits. The same constraints shape the evolution of other rapidly evolving RNA viruses; this strategy is therefore transferable well beyond SARS-CoV-2.

## 2. Results

### 2.1. Mutation-Frequency-Guided Design of Nested RT-PCR for Biotinylated PCR Products and Pyrosequencing Analysis (PSQ)

**Figure 1:**
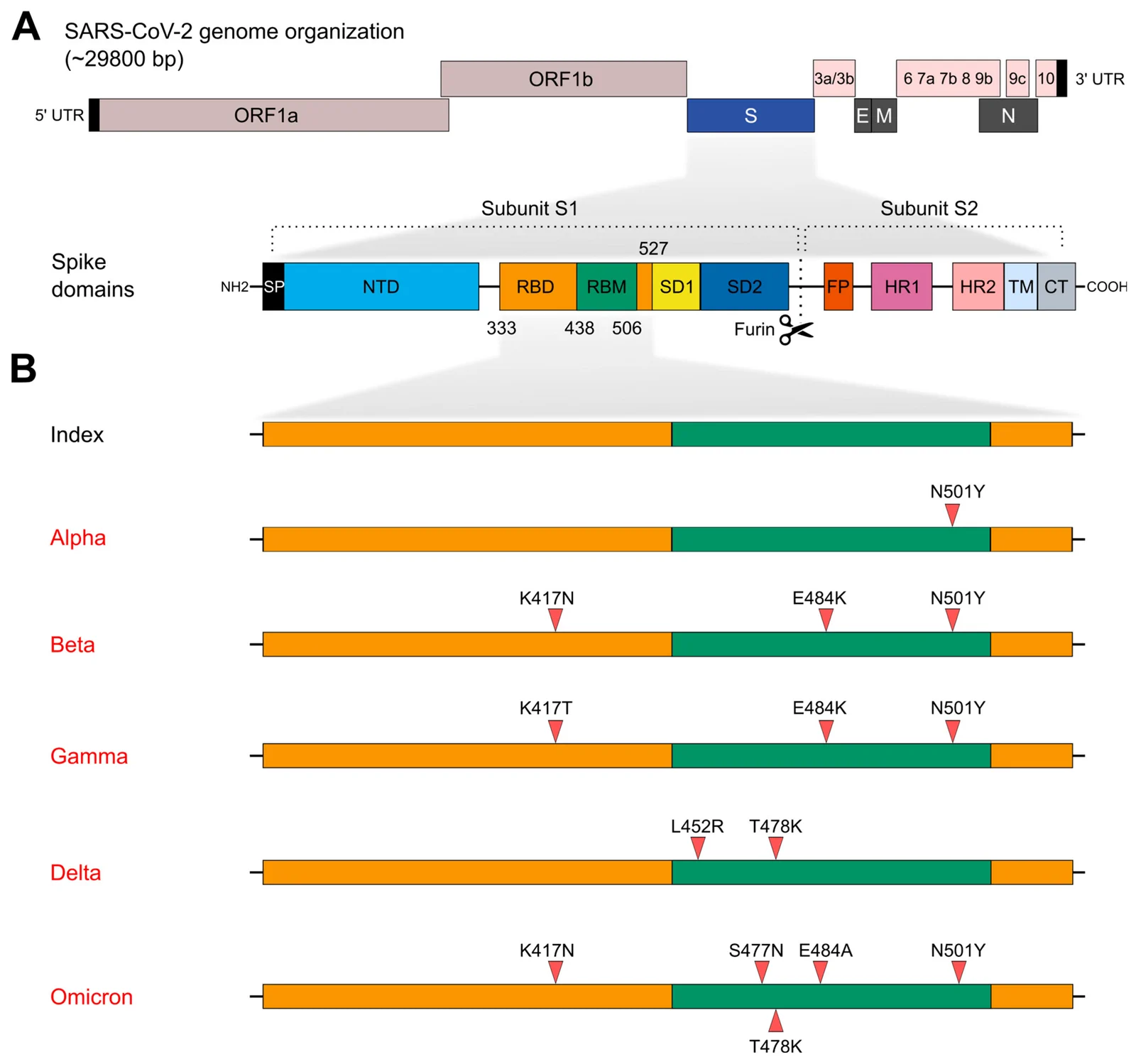
Schematic overview of the SARS-CoV-2 genome and spike gene regions harboring characteristic substitutions of variants of concern (VOCs) and variants of interest (VOIs). (**A**) Schematic representation of the SARS-CoV-2 genome showing all major open reading frames (ORF1a/1b, S, E, M, N, and accessory genes). The spike (S) gene is shown in an enlarged view below, illustrating the organization of its functional domains, including the signal peptide (SP), N-terminal domain (NTD), receptor-binding domain (RBD), receptor-binding motif (RBM), subdomains 1 and 2 (SD1/SD2), fusion peptide (FP), heptad repeats (HR1/HR2), transmembrane region (TM), and cytoplasmic tail (CT). A scissors symbol indicates the position of the furin-cleavage site between S1 and S2. (**B**) Linear representation of a selected region of the spike gene showing characteristic amino acid substitutions (“mutations of interest”) associated with early SARS-CoV-2 VOCs. For the Alpha, Beta, Gamma, Delta, and Omicron variants, substitutions relative to the Index strain are indicated by red triangles and corresponding amino-acid positions. This comparative view shows the concentration of characteristic substitutions within this spike region, which informed the selection of PSQ target fragments.

**Figure 2:**
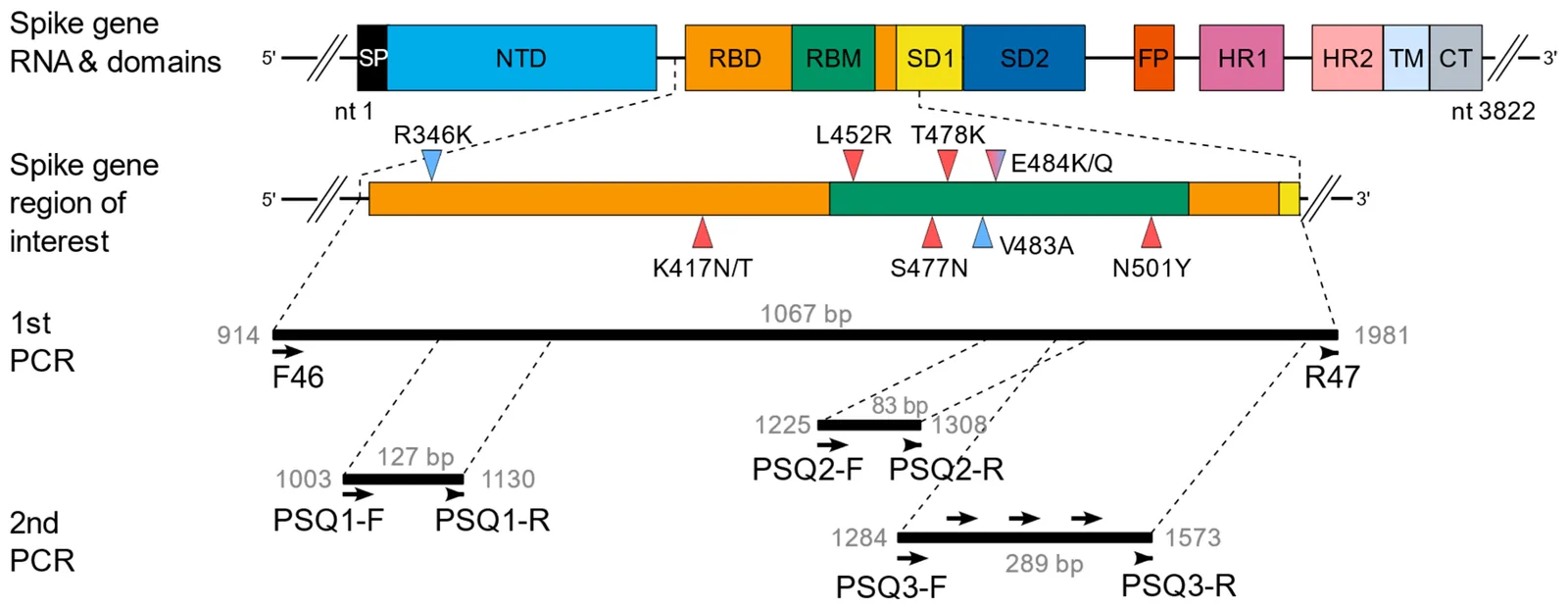
**Nested PCR-PSQ strategy targeting mutation-bearing regions of the SARS-CoV-2 spike gene.** Schematic representation of the nested PCR-PSQ strategy used to amplify and analyze defined regions of the SARS-CoV-2 spike gene. The spike gene and its major functional domains are shown at the top. A corresponding portion of the spike coding sequence is shown at higher resolution, with amino acid substitutions identified in historical VOCs highlighted. A first-round PCR amplifies a 1068 bp spike fragment, from which three nested PCRs generate shorter amplicons surrounding sites of mutations of interest (e.g., R346, K417, E484, N501). These nested amplicons form the templates for subsequent pyrosequencing to determine the nucleotide identity at selected spike positions. PSQ primers are indicated by arrows.

To identify appropriate targets for pyrosequencing-based mutation screening, we concentrated on spike protein substitutions that were both compatible with short-amplicon PSQ and epidemiologically relevant in Germany at the time of assay development. A large national SARS-CoV-2 sequencing dataset was analyzed to assess mutation frequencies and identify recurrent, lineage-defining substitutions that were common among German variants of concern in the pre-Omicron period (Section 4.7). This analysis identified recurrent, lineage-associated substitutions concentrated within the receptor-binding domain and provided a basis for selecting three target regions for the PSQ assay (Figure 1). The resulting assay architecture comprised three nested amplicons covering the spike positions R346, K417, L452, S477, T478, V483, E484, F486, F490, and N501 (Table 1; Figure 1 and Figure 2). Table 1 summarizes the corresponding primer sequences and genomic coordinates, while Figure 2 illustrates the overall nested PCR-PSQ design.

To assess the temporal stability of the mutation targets selected during assay development, mutation-frequency profiles (Supplementary Table S1) were additionally compared with datasets from 2022 and from June 2023 to December 2025 (Supplementary Tables S2 and S3). The spike mutation landscape changed substantially over time, with limited overlap in characteristic mutations between the original 2021 dataset and the later surveillance period. Several mutations that were lineage-informative during early VOC circulation subsequently became widespread among Omicron-derived lineages. In particular, K417N, S477N, T478K, and N501Y were present at high frequencies across numerous lineages in the later datasets, thereby reducing their discriminatory value for mutation-based lineage screening. Conversely, the predominant amino-acid state at other targeted positions changed over time; for example, L452R declined markedly while L452W emerged in the 2023–2025 dataset, and F486P became highly prevalent in later lineages. These temporal changes illustrate that mutation-focused screening panels require periodic reassessment and adaptation to the contemporary variant landscape.

### 2.2. Validation of the Nested PCR-PSQ Workflow

**Figure 3:**
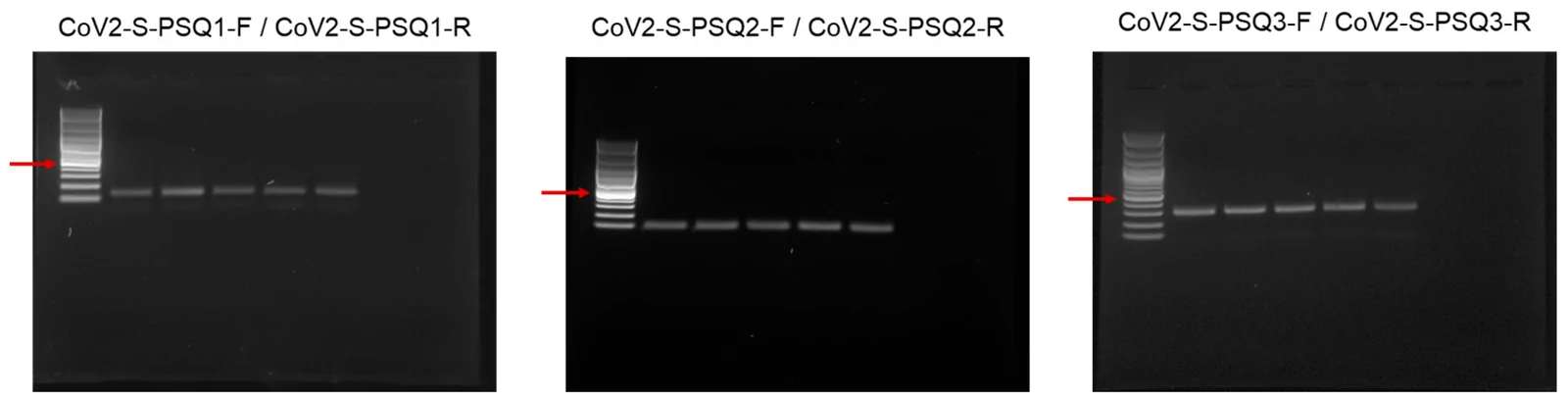
**Agarose-gel validation of nested PCR amplicons for pyrosequencing.** Representative electrophoresis images of PCR products generated with nested primer pairs CoV2-S-PSQ1-F/R, CoV2-S-PSQ2-F/R, and CoV2-S-PSQ3-F/R are shown in the **left**, **middle**, and **right** panels, respectively. PCR products were separated on 1.5% agarose gels stained with GelRed. For each nested PCR, a distinct band was observed demonstrating the expected amplicon size, with no evidence of non-specific amplification. The marker ladder has a 100 bp gradient. The 500 bp band is marked with an arrow.

Each nested amplicon exhibited a distinct, robust band without visible non-specific products or primer-dimers on 1.5% agarose gel electrophoresis, confirming the specificity and robustness of the amplification prior to PSQ (Figure 3).

All validation controls yielded negative results in pyrosequencing analysis, confirming the absence of non-specific binding.

### 2.3. Pyrosequencing Profiles of Targeted Spike Positions

**Figure 4:**
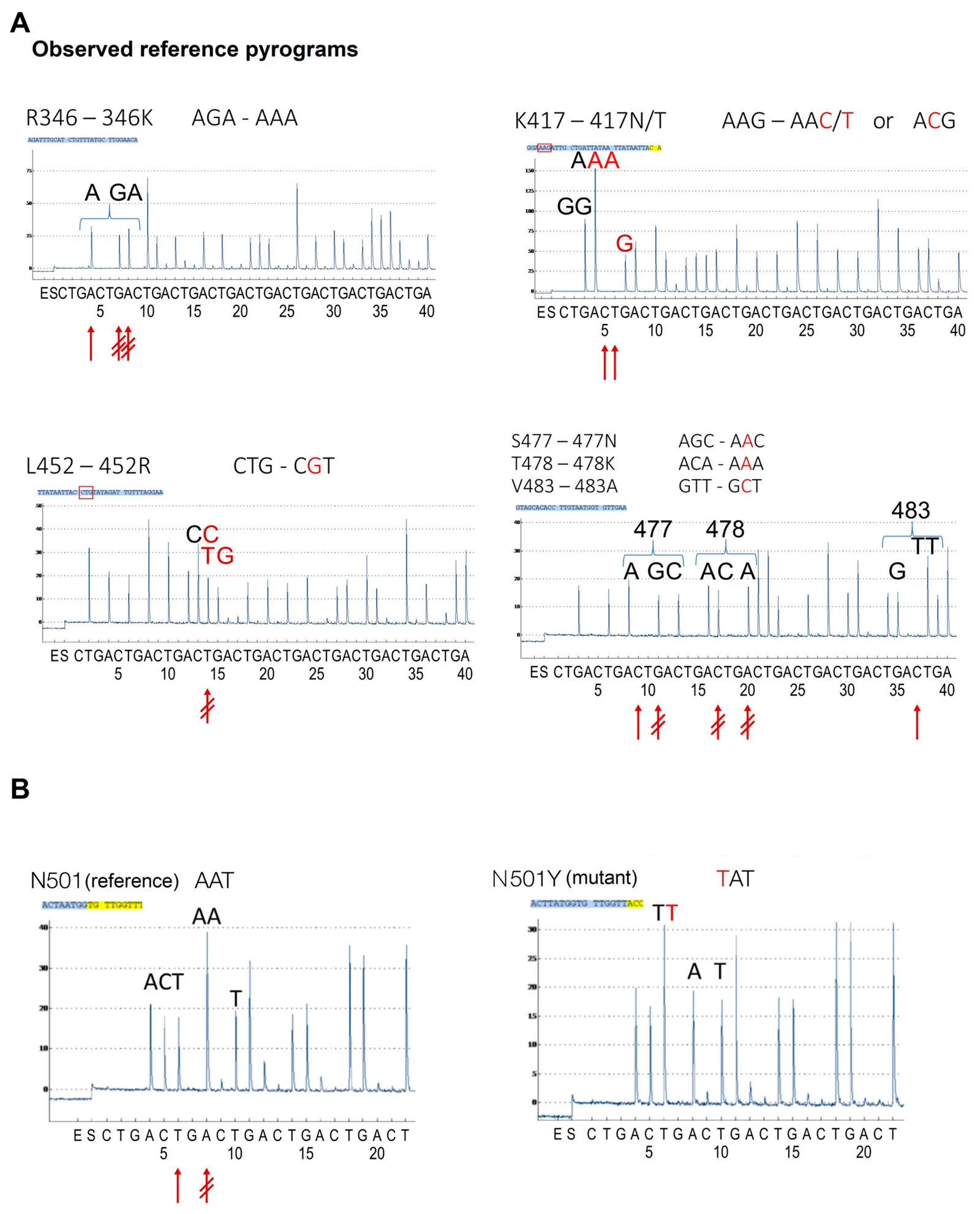
**Representative pyrograms of the PCR-PSQ assay for selected SARS-CoV-2 spike gene codons.** (**A**) Representative pyrograms of reference sequences at spike positions R346, K417, L452, S477, T478, and V483. The corresponding reference and selected mutant codons are indicated above the pyrograms, with altered nucleotides highlighted in red. Red arrows indicate selected nucleotide dispensations at which increased incorporation is expected for the indicated mutant sequence relative to the reference sequence, whereas crossed red arrows indicate dispensations at which reduced or absent incorporation is expected. Blue brackets highlight the interrogated sequence regions where indicated. (**B**) Representative pyrograms of the reference codon N501 (AAT) and the mutant N501Y (TAT), illustrating the differences in nucleotide incorporation patterns. The red arrows beneath the reference pyrogram indicate the expected changes associated with N501Y, as illustrated by the corresponding mutant pyrogram.

Representative pyrograms obtained from the nested PCR-PSQ assays are shown in Figure 4. For each interrogated spike codon, pyrosequencing of the nested amplicons yielded the expected incorporation pattern for both the reference sequence and specimens carrying VOC-associated substitutions. Differences in nucleotide incorporation patterns between wild-type and mutant codons were observed at the nucleotide positions corresponding to the interrogated base. Substitutions such as N501Y (AAT → TAT), E484K (GAA → AAA), R346K (AGA → AAA), L452R (CTG → CGT), and K417N/T (AAG → AAC/AAT/ACG) produced altered peak patterns consistent with the underlying codon changes. Comparable discrimination was obtained for substitutions at positions 477, 478, 483, 486, and 490 within the third nested amplicon. The pyrosequencing signals thereby allowed direct determination of the nucleotide identity at each position targeted by the PSQ assay.

### 2.4. Clinical Evaluation of the Nested PCR-PSQ Assays in ARI Sentinel Specimens

A total of 175 SARS-CoV-2–positive samples obtained through the national ARI sentinel system were included in the clinical evaluation of the nested PCR-PSQ assays (Ct median 28, IQR 24–33). WGS lineage assignments were available for 144/175 (82%) samples and comprised Alpha (n = 81), Beta (n = 5), Delta (n = 19), and other contemporaneous lineages (n = 39; e.g., B.1.177 and B.1.258); 31/175 (18%) samples had no lineage assignment available (e.g., not sequenced or not assignable), reflecting routine surveillance conditions. In addition, raw WGS data could be retrieved for 112 samples. Because assay development proceeded iteratively and specimen material was limited, individual samples were tested with variable PSQ targets rather than uniformly with the full panel.

Across all targets that were run, the PSQ assays generated evaluable base calls for the majority of reactions, with occasional non-evaluable results. PSQ performance per interrogated position is summarized in Table 2: evaluability 92% at position 346 (45/49) to 100% at position 452 (49/49). Median Ct values for evaluable reactions were typically in the high 20s, whereas non-evaluable outcomes were uncommon and, for several but not all targets, associated with higher Ct values (Table 2). When evaluable, PSQ provided direct base calls at the interrogated spike positions (including sites 346, 417, 452, 477, 478, 483, 484, 486, 490, and 501), enabling mutation-focused characterization of sentinel specimens across a broad range of Ct values.

Where WGS lineage assignments were available, PSQ base calls at interrogated positions were highly consistent with the lineage-defining residues expected for the assigned lineages (510/519 evaluable site-specimen comparisons). For the 112 samples with retrievable raw WGS data, we compared PSQ results with the consensus sequence at each interrogated codon. PSQ and WGS results agreed in 489 of 496 positions assessable by both methods (98.6%), with discordant results at seven positions (N501, n = 4; E484, L452, and S477, n = 1 each). Of the 727 PSQ measurements at individual spike positions, 231 could not be included in the direct comparison: corresponding WGS data were unavailable for 171 measurements, WGS coverage was insufficient at the interrogated codon for 19 measurements, and the PSQ result was not interpretable for 41 measurements. The per-sample WGS calls and the position-wise comparison with the PSQ results are provided in Supplementary Table S4.

These results support the use of nested PCR-PSQ for the targeted detection of predefined spike substitutions in routine sentinel specimens. However, this assay was not evaluated as a standalone method for lineage classification. Since different subsets of PSQ targets were used to test individual specimens and no predefined lineage-classification algorithm was evaluated, the sensitivity, specificity, and predictive values for categorical lineage assignment cannot be reliably determined from these data.

## 3. Discussion

This study describes a rapid pyrosequencing (PSQ) assay for detecting SARS-CoV-2 spike mutations characteristic of variants of concern. The nested PCR-PSQ workflow demonstrated high analytical specificity and robust performance in real-world surveillance specimens collected through the German ARI sentinel. Because the ARI sentinel encompasses a broad spectrum of sample types and viral loads, successful assay performance in this setting underscores its operational stability.

In the context of public health surveillance, rapid mutation-focused methods may provide actionable genomic information even when full genome sequencing is not immediately available or feasible [9,32,33]. The assay has several advantages over other rapid mutation detection methods. The nested design generates short amplicons suitable for targeted pyrosequencing. However, because the workflow depends on successful amplification of a 1068 bp first-round product, the present assay should not be assumed to provide a general advantage for highly fragmented starting material. In addition, PSQ provides sequence-level resolution at individual nucleotide positions, enabling precise identification of mutation patterns without requiring extensive mutation-specific probe redesign. This feature distinguishes PSQ from purely probe-based SNP assays and is consistent with its established use as an accurate and flexible platform for SNP genotyping and targeted mutation detection [25,34,35,36,37]. The principal contribution of the present study is therefore not the use of pyrosequencing *per se*, which has previously been applied to the detection of SARS-CoV-2 variants [25,34,35,36,37], but its implementation as a surveillance-oriented, mutation-focused workflow. The approach combines data-driven target selection with evaluation in routine national sentinel specimens and retrospective assessment of how the epidemiological informativeness of the selected targets changed during subsequent viral evolution.

Although PSQ cannot replace whole-genome sequencing, it is well suited as a complementary tool within a tiered surveillance strategy. Same-day mutation information can support earlier recognition of shifts in variant circulation and help prioritize samples for sequencing. This is particularly valuable during periods of high incidence, when sequencing capacity may be limited, and rapid triage of samples is essential. WHO guidance likewise emphasizes the public health value of timely and representative genomic surveillance, including integration with sentinel systems for acute respiratory infection surveillance [9,38].

A further strength of the approach is its adaptability to newly emerging SARS-CoV-2 variants. The present assay was designed around mutation constellations characteristic of earlier variants of concern and therefore represents a proof-of-concept for targeted PSQ-based surveillance rather than a fixed diagnostic solution. As SARS-CoV-2 continues to evolve, some loci included in the original design may lose discriminatory power or no longer adequately distinguish currently circulating lineages. Although the modular design allows target positions and corresponding primer sets to be adapted, application to contemporary lineages would require reassessing informative mutations and primer-binding regions. This would be followed by validating the modified assay. The original primer panel was not evaluated for compatibility with later Omicron-derived lineages. Such reassessment is particularly important in cases of recurrent convergent evolution, where identical or similar spike mutations may arise independently in different lineages, complicating the interpretation of fixed target panels [39,40].

A key limitation of the approach is its restriction to predefined genomic loci. As a targeted method, PSQ cannot capture the full genetic diversity of circulating viruses and may miss novel or unexpected mutations outside the selected regions. In addition, convergent evolution may generate mutation patterns not uniquely associated with any single lineage, thereby reducing the discriminatory value of mutation-based typing alone. These limitations reinforce the need to interpret PSQ results alongside broader genomic surveillance and, where necessary, confirmatory whole-genome sequencing [9,32,39,40]. This was also evident in the retrospective mutation-frequency analysis presented here: several targets that distinguished early VOCs subsequently became widespread among Omicron-derived lineages, whereas alternative substitutions emerged at some of the same spike positions, emphasizing the need for periodic data-driven reassessment of targeted screening panels. As with other nested PCR approaches, the additional amplification step increases the risk of carry-over contamination and therefore requires stringent separation of pre- and post-amplification procedures and appropriate negative controls.

Importantly, the methodological principles established here are not restricted to SARS-CoV-2. Many emerging and re-emerging viruses accumulate recurrent mutations associated with transmissibility, antigenicity, receptor usage, pathogenicity, or antiviral resistance. Targeted sequence-based mutation detection is therefore relevant to broader surveillance applications, including monitoring antiviral resistance in other respiratory viruses. Similar mutation-focused approaches are already well established in influenza research, where resistance-associated substitutions are monitored because they may affect antiviral susceptibility while retaining viral fitness and transmissibility [21,23,24,34,36].

Wastewater surveillance provides another example of mutation-focused monitoring. Previous studies have demonstrated the use of genomic approaches for tracking SARS-CoV-2 variants in wastewater [41,42,43,44], including targeted detection using digital RT-PCR and multiplex mutation assays [17,45,46]. However, the applicability of our PSQ workflow to wastewater remains untested and cannot be inferred from the present results, particularly given the requirement for a 1068 bp first-round amplicon, potential RNA fragmentation, and the presence of mixed viral populations.

The workflow presented in this study therefore illustrates how early genomic knowledge can be translated into adaptable, high-throughput mutation assays suitable for integration into surveillance systems. Such approaches will remain useful complements to whole-genome sequencing, particularly where rapid turnaround and scalability are required [9,38].

## 4. Materials and Methods

### 4.1. Clinical Specimens (ARI Sentinel)

Surveillance and virological characterization of respiratory viruses were conducted using clinical specimens from ambulatory patients participating in Germany’s national sentinel system for monitoring acute respiratory infections [23,27,28,47]. The specimens included in this evaluation were collected between 1 December 2020 and 13 January 2022 and were sent to RKI, where they were tested for SARS-CoV-2, influenza, and other respiratory viruses using multiplex RT-PCR assays. The ARI sentinel surveillance program was approved by the Ethics Committee of Charité-Universitätsmedizin Berlin, Germany (approval number EA2/126/11, approved 7 March 2014). The study was conducted in accordance with the Declaration of Helsinki. Written informed consent for participation in sentinel surveillance was obtained from all patients. All analyses were performed using pseudonymized data. The clinical evaluation comprised 175 specimen-level PSQ testing records. One laboratory identifier was associated with two separate sets of PSQ results. The reason for this duplication could not be determined retrospectively, and both sets of results were retained in the analysis.

### 4.2. Sample Preparation, RNA Extraction, cDNA Synthesis and Respiratory Virus Detection

Upper respiratory tract specimens, primarily nasal or oropharyngeal swabs, were processed as described previously [29,48]. Briefly, 3 mL of cell culture medium (minimum essential medium supplemented with HEPES buffer and penicillin/streptomycin) was added to the swabs to release the collected material. Total RNA was extracted from 200 µL of sample material using the MagNA Pure 96 DNA and Viral NA Small Volume Kit on a MagNA Pure 96 instrument and eluted in 50 µL. For cDNA synthesis, 25 µL of RNA was reverse-transcribed in a total reaction volume of 40 µL using random hexamer primers and 200 U Moloney murine leukemia virus reverse transcriptase (M-MLV; Thermo Fisher Scientific, Waltham, MA, USA). Reverse transcription was performed at 42 °C for 5 min, 37 °C for 30 min, and 95 °C for 5 min. The resulting cDNA was diluted 1:1 with nuclease-free water before downstream PCR analysis.

Routine detection of respiratory viruses, including SARS-CoV-2, was performed using the modular real-time PCR system established for the German ARI sentinel, as described previously [29,48]. Reactions were run on LightCycler 480 II instruments (Roche, Basel, Switzerland) in a total volume of 20 µL using 5 µL of diluted cDNA. Thermocycling consisted of an initial 5 min at 95 °C followed by 45 cycles of 95 °C for 15 s and 60 °C for 30 s.

### 4.3. Spike Gene PCR and Nested PSQ-PCR

Amplification of the SARS-CoV-2 spike fragments was performed by PCR in a 50 µL reaction volume containing 5 µL cDNA, 0.5 U platinum TaqPol polymerase (Invitrogen, Carlsbad, CA, USA), 2 mM MgCl2, 200 µM dNTP and 250 nM of each specific primer of the first PCR round CoV2-F46 and CoV2-R47 (TibMolBiol, Berlin, Germany, [31]). PCR was performed in an Eppendorf thermocycler under the following conditions: 3 min at 95 °C, 35 cycles of 95 °C for 3 s and 55 °C for 30 s and 1 min each at 72 °C, with a final extension step of 3 min at 72 °C.

The nested PCR was designed based on three nested amplicons, using biotinylated reverse primers: CoV2-S-PSQ1-F–CoV2-S-PSQ1-Rbio, CoV2-S-PSQ2-F–CoV2-S-PSQ2-Rbio, and CoV2-S-PSQ3-F–CoV2-S-PSQ3-Rbio, each covering one or several mutations of interest. Amplification was performed in a 50 µL reaction volume containing 2 µL template from the first PCR, 0.5 U platinum TaqPol polymerase (Thermo Fisher Scientific, Darmstadt, Germany), 2 mM MgCl2, 200 µM dNTP and 250 nM of each specific primer under following conditions: 95 °C for 15 min followed by 50 cycles of 94 °C for 30 s, annealing at 65 °C for 30 s and elongation at 72 °C for 30 s, with a final extension of 72 °C for 10 min. PCR products were analysed for validation purposes on a 1.5% agarose gel, stained with gel red, UV-illuminated, and stored at 4 °C.

The assay design was built on a previously published first-round RT-PCR protocol developed at the University of Geneva [31]. Amplification of cDNA with primers CoV2-F46 and CoV2-R47 produced a 1068 bp fragment spanning the entire RBD of the SARS-CoV-2 spike gene. Based on this outer PCR, three nested PCR assays were designed in-house to generate short, biotinylated amplicons suitable for downstream pyrosequencing.

Nested primer design was based on a consensus sequence derived from an alignment of representative SARS-CoV-2 spike sequences together with the reference strain hCoV-19/Wuhan/WIV04/2019 (WIV04; GISAID accession number EPI_ISL_402124) and was performed using the PyroMark^TM^ Assay Design Software version 2.0.2.5 (Qiagen, Hilden, Germany). Each nested PCR employed a biotinylated antisense primer to permit strand immobilization for pyrosequencing. For real-time sequencing with PSQ, specific sequencing primers were designed and positioned in close proximity to the targeted nucleotide positions, enabling accurate detection of substitutions within each amplified region. Table 1 and Figure 2 provide an overview of all primers used in this study, including their sequences, spike gene coordinates relative to the index strain, and the corresponding amplicon sizes.

Primer and template sequences were subjected to rigorous examination to exclude any potential complementary formations, such as primer-dimers and self-complementary structures, including hairpins. PCR conditions, including magnesium concentration and annealing temperature, were optimized using cDNA from control samples and, for nested PCR conditions, the outer PCR fragment as template, respectively.

All pre- and post-amplification procedures were performed in physically separated work areas using dedicated equipment and aerosol-resistant filter tips. No-template controls were included in each PCR run.

### 4.4. Pyrosequencing

Pyrosequencing analyses were carried out with the Pyromark Q96 System (QIAgen Hilden) [22,23]. A total of 20 µL of each biotinylated nested PCR product was immobilized on 4 µL of Streptavidine Sepharose beads, 16 µL H_2_O and 40 µL binding buffer for 10 min at room temperature with 5 min shaking at 1000 rpm. Using the PyroMark Vacuum Prep Workstation, samples were washed in H_2_O for approximately 20 s. The Vacuum Prep Tool was immersed in 70% ethanol for 5 s, followed by washing with denaturant and washing buffer for 5 s each for DNA single-strand preparation.

The beads were transferred to a PSQ HS 96 plate prefilled with 0.625 µM sequencing primer in 39 µL annealing buffer per well. For primer annealing, the sample plate was heated at 80 °C for 2 min and then cooled to room temperature. Pyrosequencing analysis of the immobilized strand was performed with up to 96 samples in parallel at 28 °C using the PyroMark^TM^ ID instrument (Biotage, Uppsala, Sweden) and 96 PyroMark enzyme and substrate mixture (Qiagen, Hilden).

For analytical validation, the following controls were included for each nested amplicon: (1) PCR no-template control combined with one sequencing primer; (2) sequencing primer only; (3) biotinylated primer combined with one sequencing primer (no PCR amplification); (4) biotinylated primer only; and (5) template only (without primers or amplification).

### 4.5. Next Generation Sequencing

SARS-CoV-2 whole-genome sequencing was performed using previously described tiled-amplicon sequencing workflows [2]. For Illumina sequencing, libraries were generated using the CleanPlex SARS-CoV-2 amplicon panel (Paragon Genomics, Fremont, CA, USA) and sequenced on an iSeq 100 system (Illumina, San Diego, CA, USA) using 150 bp paired-end reads. For Oxford Nanopore sequencing, libraries were generated using the NEBNext ARTIC SARS-CoV-2 Companion Kit with ARTIC primer sets and sequenced on MinION or GridION instruments (Oxford Nanopore Technologies, Oxford, UK). Consensus genomes were reconstructed using covPipe for Illumina data and poreCov for Nanopore data, as described previously [2]. For harmonized quality control and lineage assignment of the 2021 sequence dataset, consensus sequences were processed using poreCov in FASTA mode. Sequences were compared with the SARS-CoV-2 reference genome NC_045512.2 using a minimum sequence-similarity threshold of 90%, and genomes containing more than 5% ambiguous N bases were excluded. PANGO lineages were assigned using Pangolin v3.1.17 with PangoLEARN release 6 December 2021, as described previously [2].

For the direct comparison of PSQ and WGS results, available Nanopore sequencing data were reprocessed using poreCov v1.12.5 against the reference genome of the index strain (MN908947.3). The consensus codon corresponding to each PSQ-interrogated spike position was extracted and translated to the amino-acid level. A WGS position was considered non-assessable if any nucleotide of the respective codon was masked as N because of insufficient sequence information or contained an ambiguous IUPAC base. Only positions with evaluable calls by both PSQ and WGS were included in the direct concordance analysis.

### 4.6. Agarose Gel Electrophoresis

The PCR fragments used for sequence determination were analysed by agarose gel electrophoresis. The agarose gels had a concentration of 1.5% (*w*/*v*) in 1× TAE buffer (400 mM Tris, 500 mM sodium acetate, 10 mM EDTA and GelRed; pH 7.9). Five microlitres of the PCR product were mixed with two microlitres of 6× loading dye (30% glycerol [*v*/*v*], 50 mM EDTA, 0.15% bromophenol blue [*w*/*v*], 10× TAE) prior to application to the gel slots. The PCR products were separated by electrophoresis at 80–100 V (4–5 V/cm) for 30 min. Visualization of the PCR products was carried out using UV light (λ 366 nm). A ready-to-use DNA ladder (O’Range Ruler™ 100 bp DNA Ladder) was used to detect the molecular weight of the amplicons.

### 4.7. Mutation Frequency Analysis

Lineage-specific mutation frequencies were quantified using the SARS-CoV-2 Mutation Frequency Calculator (SC2MFC version 0.0.4; https://github.com/rki-mf1/sc2-mutation-frequency-calculator; accessed on 26 October 2026). Mutation profiles derived from DESH (German Electronic Sequence DataHub for SARS-CoV-2 genomes, available at https://doi.org/10.5281/zenodo.17880354) were exported via covSonar (https://github.com/rki-mf1/covsonar, version 1.1.11; accessed on 26 October 2026) from 1 January 2021 to 30 November 2021. SC2MFC calculated, for each genome represented in the dataset (n = 168,218), the proportion of genomes carrying each nucleotide or amino-acid change and assembled a lineage-by-mutation frequency matrix. This pre-Omicron time frame was chosen to reflect the period when we identified the signature mutations for primer design. The tool supports a minimum-sample filter per lineage (default: 10 genomes) and a user-defined frequency cutoff (default: 75%) to focus on high-frequency, characteristic lineage-associated mutations; we applied these filters using their defaults. Lineages that did not meet the minimum-sample filter were aggregated into an “other lineages” category and used as a pooled background when assessing candidate mutations. The resulting matrix, available as Supplementary Table S1, was screened for abundant (“characteristic”) mutations. Candidate mutations were cross-checked against WHO/ECDC lists and the literature to select mutations relevant to primer design. Algorithmic details and validation of SC2MFC are described in the associated master’s thesis [49].

Using the same procedure, two additional time frames were also analyzed to evaluate the ongoing validity of the selected signature mutations. SC2MFC were used on mutation profiles extracted from SARS-CoV-2 genomes between 1 January 2022 and 31 December 2022 (“main Omicron time”) and 1 June 2023 and 11 December 2025 (“current time with down-scaled genomic surveillance”). The resulting mutation frequency tables are available in the Supplementary Tables S2 and S3.

## 5. Conclusions

A nested PCR-Pyrosequencing assay for rapid detection of SARS-CoV-2 variant-associated spike mutations was designed, and its performance was validated in population-based surveillance samples. The assay showed high analytical specificity, reliable performance across a range of viral loads, and strong agreement with whole-genome sequencing. These findings support targeted PSQ as a rapid, complementary tool for monitoring predefined, epidemiologically relevant mutations within multi-level surveillance systems, while WGS remains essential for the comprehensive characterisation and discovery of newly emerging variants.

Beyond SARS-CoV-2, this workflow provides a generalizable template for developing short-amplicon, mutation-focused assays for emerging viruses. As public health systems prepare for future epidemics and pandemics, rapid and adaptable molecular surveillance tools will remain essential components of early detection and response.

## Figures and Tables

**Table 1:** Outer PCR [31], nested PCR and PSQ primers used to amplify and sequence SARS-CoV-2 spike regions with VOC-associated amino-acid substitutions.

Primer	Sequence (5′-3′)	Position ^1^	Target Codons
CoV2-spike_F46	CCTTCACTGTAGAAAAAGGAATC	914–936	outer PCR, all positions, R346-N501
CoV2-spike_R47	CATATGAGTTGTTGACATGTTCAG	1958–1981	outer PCR, all positions, R346-N501
CoV2-S-PSQ1-F	TTGTGCCCTTTTGGTGAAGTTTT	1003–1025	R346
CoV2-S-PSQ1-Rbio	AAAGTGGAAAATGATGCGGAATTA	1107–1130	R346
CoV2-S-PSQ1-Seq	AGTTTTTAACGCCACC	1020–1035	R346
CoV2-S-PSQ2-F	CAAATCGCTCCAGGGCAAAC	1225–1244	K417
CoV2-S-PSQ2-Rbio	CCAAGCTATAACGCAGCCTGTAAA	1285–1308	K417
CoV2-S-PSQ2-Seq	GCTCCAGGGCAAACT	1231–1245	K417
CoV2-S-PSQ3-F	TTTTACAGGCTGCGTTATAGCTT	1284–1306	L452-N501
CoV2-S-PSQ3-Rbio	AAACAGTTGCTGGTGCATGTA	1553–1573	L452-N501
CoV2-S-PSQ3-Seq1	TCTAAGGTTGGTGGTAA	1327–1343	L452
CoV2-S-PSQ3-Seq2	GAAATCTATCAGGCCG	1411–1427	S477-N490
CoV2-S-PSQ3-Seq3	CACCTTGTAATGGTGT	1433–1448	E484
CoV2-S-PSQ3-Seq4	CATATGGTTTCCAACCC	1481–1497	N501

^1^ based on the index strain hCoV-19/Wuhan/WIV04/2019 (EPI_ISL_402124). F, forward primer; Rbio, 5′-biotinylated reverse primer; Seq, pyrosequencing primer. PSQ1–PSQ3 denote the three nested PCR amplicons used for downstream pyrosequencing.

**Table 2:** Evaluability of PSQ targets in ARI sentinel specimens ^1,2^.

Spike Site (AA Position)	PSQ Tests, n	Evaluable, n (%)	Non-Evaluable, n (%)	Median Ct (Evaluable)	Median Ct (Not Evaluable)
R346K	49	45 (92)	4 (8)	27	36
K417N	48	46 (96)	2 (4)	28	24
L452R	49	49 (100)	0 (0)	28	n.a.
S477N	49	47 (96)	2 (4)	28	30.5
T478K	49	46 (94)	3 (6)	27.5	31
V483A	49	46 (94)	3 (6)	27.5	31
E484K/Q	175	167 (95)	8 (5)	28	24
F486K/I/Y/S/L	49	46 (94)	3 (6)	28	25
F490L/S	49	46 (94)	3 (6)	28	25
N501Y	161	148 (92)	13 (8)	28	29

^1^ “Evaluable” indicates an interpretable PSQ readout at the interrogated position. Tested samples without an interpretable call were counted as non-evaluable. Percentages are relative to PSQ tests performed. Ct values are medians (rounded to whole numbers). ^2^ The corresponding WGS-derived nucleotide codons and amino-acid residues, together with individual PSQ calls and WGS lineage assignments, are provided in Supplementary Table S4.

## Data Availability

The mutation-frequency data generated and analyzed in this study are provided in Supplementary Tables S1–S4. Mutation profiles used as input for the SARS-CoV-2 mutation-frequency analyses were derived from the German Electronic Sequence DataHub (DESH) and are available through the archived dataset at Zenodo (https://doi.org/10.5281/zenodo.17880354). Mutation-frequency analyses were performed using the SARS-CoV-2 Mutation Frequency Calculator (SC2MFC, version 0.0.4), which is publicly available at https://github.com/rki-mf1/sc2-mutation-frequency-calculator (accessed on 26 October 2026).

## Supplementary Materials

The following supporting information can be downloaded at: https://www.mdpi.com/article/10.3390/ijms27219769/s1.
